# Prevalence of celiac disease in a Tunisian cohort

**DOI:** 10.3389/fgstr.2025.1619533

**Published:** 2025-10-20

**Authors:** Mariam Ghozzi, Marco Kai, Tanja Seifert, Sarra Melayah, Fatma Mechi, Sarra Romdhani, Zeineb Ben Chedly, Ibtissem Ghedira

**Affiliations:** ^1^ Laboratory of Immunology, Farhat Hached University Hospital, Sousse, Tunisia; ^2^ Faculty of Pharmacy, Department of Immunology, University of Monastir, Monastir, Tunisia; ^3^ Research Laboratory for “Epidemiology and Immunogenetics of Viral Infections” (LR14SP02), Sahloul University Hospital, University of Sousse, Sousse, Tunisia; ^4^ Institute for Experimental Immunology, Affiliated with EUROIMMUN Medizinische Labordiagnostika AG, Luebeck, Germany; ^5^ LR12SP11, Biochemistry Department, Sahloul University Hospital, Sousse, Tunisia

**Keywords:** celiac disease, autoantibodies, tissue transglutaminase, deamidated gliadin peptides, indirect immunofluorescence, ELISA, immunoblot, Tunisia

## Abstract

**Background:**

Accurate and non-invasive diagnostics of celiac disease are essential for effective patient management. Although small intestine biopsy remains the diagnostic gold standard, serological assays offer a promising alternative. This study evaluated the performance and concordance of immunoblot, indirect immunofluorescence test (IIFT), and enzyme-linked immunosorbent assay (ELISA) in detecting celiac disease-specific autoantibodies in a Tunisian cohort, aiming to assess the potential of combining various assays to reduce reliance on invasive procedures.

**Methods:**

Serum samples from 80 celiac disease patients and appropriate controls were analyzed using three serological methods. IIFT was employed to detect IgA autoantibodies against endomysium using primate liver and human umbilical cord substrates. ELISA was used to quantify anti-tissue transglutaminase (tTG) IgA and deamidated gliadin peptide (DGP) autoantibodies. Immunoblots assessed additional autoantibodies (tTG, GAF-3X, and ASCA), along with further evaluation of IgG autoantibodies (intrinsic factor and parietal cell antibodies). Concordance among methods was evaluated.

**Results:**

IIFT detected anti-endomysium IgA autoantibodies in 100% (80/80) of celiac patients (in this cohort), with no positivity in controls. ELISA demonstrated that both tTG IgA and DGP autoantibodies were present in all celiac disease patients. All controls (n = 158) were ELISA-negative, indicating 100% specificity in both assays. Immunoblots revealed tTG IgA in 99% (79/80) of patients, while GAF-3X autoantibodies were detected in 94% (IgA) and 85% (IgG) of celiac patients. In addition, ASCA IgA autoantibodies were present in 31% of celiac disease patients, with minimal reactivity observed in controls. A Venn diagram illustrated high concordance among the assays for tTG autoantibody detection, reinforcing the reliability of this autoantibody marker.

**Conclusion:**

The robust and consistent detection of celiac disease-specific autoantibodies, particularly tTG IgA and DGP autoantibodies, across multiple serological platforms underscores their diagnostic utility. The high concordance among these markers supports the potential of combined autoantibody testing to serve as a non-invasive alternative to biopsy, thereby enhancing clinical management of celiac disease.

## Introduction

Accurate and timely diagnosis of autoimmune and infectious diseases is crucial for effective treatment and management (1). Among the various diagnostic tools available, immunoblots, indirect immunofluorescence tests (IIFT) and enzyme-linked immunosorbent assays (ELISA) are widely used due to their specificity and sensitivity. These tests play a pivotal role in detecting antibodies or antigens associated with various pathogens and autoimmune conditions. Despite their widespread use, the comparative efficacy and reliability of these diagnostic methods can vary based on the population being studied, making it essential to evaluate their performance in diverse demographic settings.

Tunisia, a North African country with a unique genetic landscape, provides an interesting population for genetic and epidemiological studies. The Tunisian population is characterized by remarkable genetic heterogeneity, with components from Europe, Sub-Saharan Africa and Asia, reflecting its rich historical background and migration patterns (2, 3). While obtaining specific prevalence data for autoimmune and infectious diseases in Tunisia would require further investigation, the country’s diverse genetic makeup makes it a potentially valuable cohort for studying various health conditions and evaluating diagnostic techniques.

Celiac disease, a chronic autoimmune disorder triggered by the ingestion of gluten in genetically predisposed individuals, is a condition where precise diagnosis is paramount (4–6). Across Middle Eastern and North African (MENA) populations, general-population screening studies estimate celiac disease prevalence at roughly 0.5-1% overall, with country-level point estimates of ~0.5% in Tunisia, ~0.53% in Egypt, ~0.79% in Libya, ~0.88% in Iran, and higher rates reported in Saudi Arabia (up to 3.2% in some screening studies (7–12). The disease is characterized by an immune-mediated response leading to inflammation and damage to the small intestine’s lining, resulting in malabsorption of nutrients (13, 14). Traditionally, diagnostics of celiac disease in Tunisia often relies on small intestine biopsy, which, despite being effective, is invasive and burdensome for patients (7, 15). Besides its invasiveness, duodenal biopsy requires endoscopy and sedation. This exposes patients to small but measurable cardiopulmonary risks and occasional complications, while also incurring nontrivial costs and depending on endoscopy capacity that remains limited in many low- and middle-income settings (16–19).

Diagnostic tests for celiac disease, such as blot, IIFT, and ELISA, are crucial for identifying the presence of specific antibodies (20, 21). These autoantibodies include anti-tissue transglutaminase (tTG), which is the primary marker for celiac disease; deamidated gliadin peptides (GAF-3X); anti-*Saccharomyces cerevisiae* antibodies (ASCA); parietal cell antibodies (PCA); and intrinsic factor antibodies (IF) (22). Accurate detection of these antibodies is essential for confirming a celiac disease diagnosis and to allow appropriate dietary management. The primary aim of this study is to evaluate the potential of using a combination of these non-invasive assays to reduce the reliance on biopsies for diagnosing celiac disease.

This study aims to evaluate and compare the results of blot, IIFT, and ELISA testing in a Tunisian cohort, specifically focusing on patients diagnosed with celiac disease. By analyzing the sensitivity and specificity of each method, we seek to determine the most reliable and effective combination of diagnostic tools that can reduce the need for invasive biopsies. Furthermore, this research will contribute to the broader understanding of how these diagnostic methods perform in different demographic contexts, potentially improving better clinical practice and health policies.

Through a comprehensive evaluation of these diagnostic methods, this study aims to provide valuable insight that can enhance the clinical management of celiac disease and other autoimmune and infectious diseases in Tunisia and similar settings.

## Materials and methods

### Cohorts and samples

This study examined anonymized sera of 80 patients diagnosed with celiac disease (77.5% females; mean age 32 years (18–65 years)) and 78 patients with non-celiac gastrointestinal complaints (disease control; 77.5% females; mean age 40.6 years (18–78 years)), as well as healthy controls (n=80, mean age 34.91) (**Table 1**). Diagnostics were performed according to the guideline of *The European Society for the Study of Coeliac Disease* (23). All participants with positive serology underwent esophagogastroduodenoscopy with multiple duodenal biopsies to confirm celiac disease. Controls (all serology-negative) did not undergo biopsy. Sera were transferred on dry ice and stored at -80 °C before analysis.

**Table 1:** Characteristics of celiac disease patients and disease controls.

Panel	Sample size	Mean Age ± SD	Women (%)
Celiac disease	80	32 ± 11.3	77.5
Disease controls	78	40.64 ± 15.94	64.1
Healthy controls	80	34.91 ± 10.8	76.2

The study was approved by the local Ethics Committee (CER: 30–2022 Committee of medical ethics and research, Farhat Hachet Hospital, Sousse). Written informed consent was obtained from each patient at enrolment.

### Diagnostic methods

#### Indirect immunofluorescence

Indirect immunofluorescence tests (IIFT) of IgA autoantibodies were performed in all three groups (celiac disease, disease controls, healthy controls) against endomysium (EMA) on monkey liver (EUROPLUS Liver (monkey), IgA, FA 1914-1A; EUROIMMUN Medizinische Labordiagnostika AG, Lübeck, Germany) and umbilical cord. For the latter, cryostat sections (4 μm thick) of human umbilical cord, prepared in Farhat Hached hospital’s laboratory of immunology, served as the substrate. Fluorescein isothiocyanate-labelled anti-human IgA antibodies were used for detection. Screening was performed at a 1:10 dilution. Reactive samples were confirmed ≥ 1:10, and a result was considered positive if the connective tissue surrounding the muscle cells exhibited bright fluorescence in a honeycomb pattern. Borderline results were retested and reviewed by another specialist; no inter-reader discrepancies were observed. Since the image acquisition setup did not support calibrated overlays, scale bars could not be rendered at acquisition. The images are provided for qualitative illustration only; no morphometric measurements were performed.

#### Immunoblot

In addition, all sera of two groups (celiac disease and disease controls) were analyzed by immunoblot (EUROLINE autoimmune gastrointestinal diseases, IgG and IgA, DL 1360 A and G; EUROIMMUN Medizinische Labordiagnostika AG, Lübeck, Germany) for the detection of autoantibodies against tissue transglutaminase (tTG) and gliadin-analogue fusion peptide (GAF-3X). Mannan from *Saccharomyces cerevisiae* (ASCA) IgA (used in the differential diagnosis of Crohn’s disease) was included to contextualize assay specificity; given its limited disease specificity and occasional positivity in celiac disease, we recorded its frequency across cohorts. The immunoblot (EUROLINE autoimmune gastrointestinal diseases, IgG, DL 1360 G; EUROIMMUN Medizinische Labordiagnostika AG, Lübeck, Germany) was further used to detect intrinsic factor (IF) and parietal cells antigen (PCA).

#### ELISA

ELISA were performed in all three groups (celiac disease, disease controls, healthy controls). For the quantitative determination of anti-tissue-transglutaminase IgA autoantibodies in sera, the Orgentec ELISA kit (Orgentec Diagnostika GmbH, Mainz, Germany) was used according to manufacturer instructions. For the determination of autoantibodies against deamidated gliadin peptide (GAF-3X), the Anti-Gliadin (GAF-3X) ELISA kit (EV 3011–9601 G and A; EUROIMMUN Medizinische Labordiagnostika AG, Lübeck, Germany) was used according to manufacturer instructions.

### Data governance and analysis

Wet-lab work and primary data acquisition were conducted at Laboratory of Immunology, Farhat Hached University Hospital (Sousse) independent of the manufacturer. Statistical analyses were performed by T.S. and M.K. (Euroimmun) and independently evaluated by all other co-authors. Both commercial co-authors (Euroimmun) had no role in study design and decision to publish and did not have access to identifiable patient data. Data were held on institutional servers; only de-identified IDs were used for analysis. Samples were blinded to clinical group during analysis; group labels were revealed only after primary analyses were finalized.

### Statistical analysis

We report sensitivity, specificity, positive and negative predictive values with exact (Clopper-Pearson) 95% confidence intervals. Agreement between assays was quantified using Cohen’s κ. Receiver operating curves (ROC) were generated for assays with quantitative outputs in both cases and controls.

We investigated a panel consisting of 80 celiac disease cases, 78 disease controls and 80 healthy controls. The study was planned to estimate sensitivity and specificity with a 95% confidence interval half-width ≤5%. For the true proportion in the 0.9–1.0 range, n≈80 yields an expected half-width of ≤5% using exact binominal methods; control totals provide comparable precision.

## Results

### High sensitivity and specificity of endomysium autoantibodies

**Figure 1:**
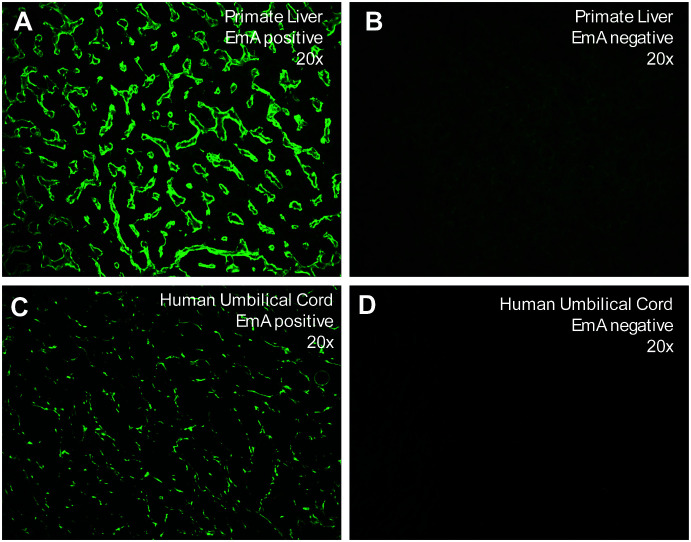
Tissue sections of primate liver **(A, B)** and human umbilical cord **(C, D)** after incubation of EMA positive **(A, C)** and EMA negative **(B, D)** patient samples. Acquired at 20X magnification. Image shown for qualitative illustration.

Using indirect immunofluorescence (IIFT) on primate liver and human umbilical cord substrates, IgA autoantibodies against endomysium were detected in 100% (80/80) of celiac disease patients, while none of the controls (disease and healthy controls) showed positivity on either substrate (**Table 2**). Representative staining patterns are shown in **Figure 1**.

**Table 2:** Comprehensive autoantibody detection rates in celiac disease patients and controls.

Panel	IIFT		EUROLINE								ELISA		
	Primate liver	Human umbilical cord	tTG IgA	GAF-3X IgA	ASCA IgA	tTG IgG	GAF-3X IgG	ASCA IgG	IF IgG	PCA IgG	tTG IgA	DGP IgA	DGP IgG
Celiac disease	**100 (80)**	**100 (80)**	**98.75 (79)**	93.75 (75)	31.25 (25)	61.25 (49)	85 (68)	42.5 (34)	1.25 (1)	25 (20)	**100 (80)**	**100 (80)**	**100 (80)**
Disease controls	**0 (0)**	**0 (0)**	**0 (0)**	5.13 (4)	8.97 (7)	0 (0)	0 (0)	2.56 (2)	0 (0)	16.67 (13)	**0** **(0)**	**0** **(0)**	**0** **(0)**
Healthy controls	**0 (0)**	**0 (0)**	–	–	–	–	–	–	–	–	**0** **(0)**	**0** **(0)**	**0** **(0)**

Bold values indicate best performance

### Robust diagnostic accuracy of anti-tissue transglutaminase autoantibodies in this cohort

**Figure 2:**
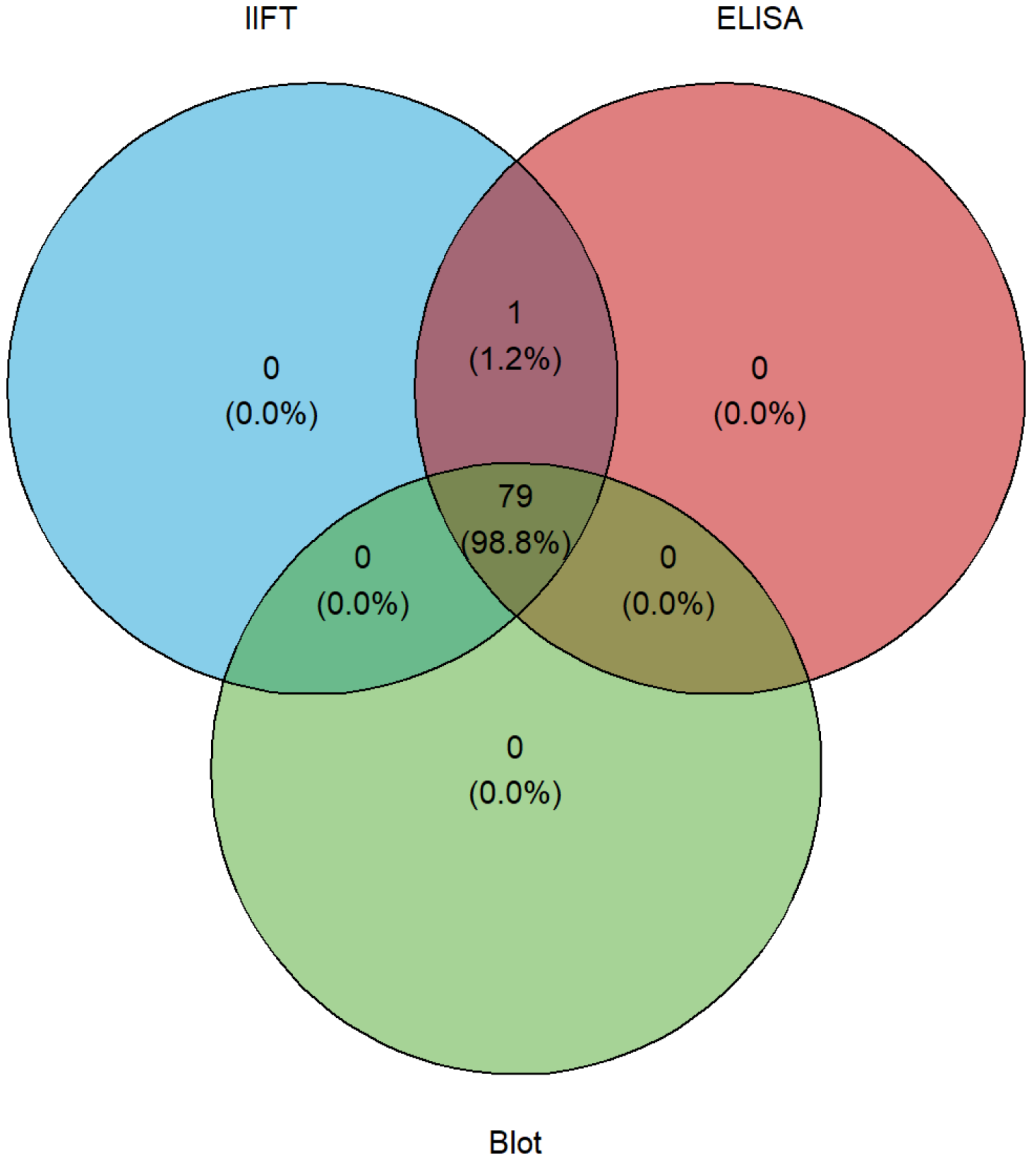
Venn diagram illustrating the overlap between IIFT, ELISA, and blot data sets.

Autoantibodies against tissue transglutaminase were measured using both ELISA and immunoblot methods. tTG IgA autoantibodies were detected in 100% (80/80) of celiac disease patients by ELISA and in 99% (79/80) by immunoblot. No tTG IgA autoantibodies were detected in disease and healthy controls (**Table 2**). Across both control groups (n=158), tTG IgA ELISA showed 100.0% specificity (97.7–100.0); combined with case data, sensitivity, PPV and NPV were each 100.0% with exact 95% CIs (95.5–100.0), (95.5–100.0) and (97.7–100.0), respectively (this cohort). ROC analysis across all participants demonstrated excellent discrimination for tTG IgA (AUC 1.0, CI 1.0–1.0) (**Supplementary Figure S1**). Using **Figure 2** illustrates the overlap between blot, IIFT, and ELISA results for tTG autoantibodies that further demonstrates the high concordance among these assays (Pearson’s = 0.962, *p* < 0.0001). Pairwise agreement across celiac disease cases and disease controls showed 157/158 concordant results for ELISA vs. blot (n=158, 99.4%, Cohen’s κ = 0.987) and IIFT vs. blot (n=158, 99.4%, Cohen’s κ = 0.987), while for ELISA vs. IIFT 238/238 concordant results (n=238, 100%, Cohen’s κ = 1.0) were observed.

### Excellent detection rates of anti-deamidated gliadin peptides (DGP/GAF-3X) autoantibodies

Using ELISA, deamidated gliadin peptide (DPG) autoantibodies were detected in 100% (80/80) of celiac disease patients, while no DGP autoantibodies were detected in disease controls (**Table 2**). ELISA data achieved 100.0% sensitivity and 100.0% specificity with exact CIs (95.5–100.0) and (97.7–100.0); PPV and NPV were 100.0% with the same CIs (this cohort). In addition, blot immunoassays showed that GAF-3X IgA autoantibodies were present in 94% (75/80) of celiac disease patients and in 5% (4/78) of disease controls, while GAF-3X IgG autoantibodies were found in 85% (68/80) of celiac disease patients. No disease control tested positive for GAF-3X IgG autoantibodies (**Table 2**). ROC analysis across all participants demonstrated excellent discrimination for DGP (AUC 1.0, CI 1.0–1.0) (**Supplementary Figure S1**).

### Moderate sensitivity of anti-*Saccharomyces cerevisiae* autoantibodies

ASCA IgA autoantibodies, tested by immunoblot, were present in 31% of celiac disease patients and 9% of celiac disease controls, whereas ASCA IgG autoantibodies were detected in 43% (34/80) of celiac disease patients and 3% (2/78) of disease controls (**Table 2**).

### Variable expression of additional IgG autoantibodies

Parietal cell antibodies (PCA) IgG autoantibodies, analyzed by immunoblot, were found in 25% (20/80) of celiac disease patients and 17% (13/78) of disease controls, while intrinsic factor (IF) IgG autoantibodies were detected in 1% (1/80) of celiac disease patients; no IF autoantibodies were observed in disease controls (**Table 2**).

## Discussion

Celiac disease diagnostics has evolved significantly in recent years, with serological tests playing an increasingly important role. This discussion will focus on the sensitivity and specificity of various serological tests for celiac disease, their potential to reduce the need for invasive biopsies, and the implications for clinical practice.

### Serological tests: sensitivity and specificity

Our study in the Tunisian cohort demonstrates remarkably high sensitivity for several serological tests used in celiac disease diagnostics. The IIFT for IgA EMA showed 100% sensitivity, aligning with the high specificity reported by Sheppard and colleagues for EMA tests (99.6% in adults) (24). This perfect sensitivity in our cohort surpasses the 88.0% sensitivity reported in their meta-analysis, potentially highlighting the effectiveness of this test in the Tunisian population. Similarly, our ELISA results for tTG and DGP showed 100% sensitivity, which is higher than the 90.7% sensitivity for tTG reported by Sheppard et al. (24). This high sensitivity aligns with the findings of Zintzaras and Germenis (21), who reported high accuracy for ELISA-based tTG tests (21). The blot analysis in our study showed slightly lower but still impressive sensitivity, with 99% of celiac disease patients testing positive for IgA autoantibodies against tTG. This high sensitivity, combined with the absence of false positives in the disease control group, suggests that the blot test could be a reliable diagnostic tool for celiac disease in this population.

### Comparative performance of diagnostic methods

Our results indicate that IIFT and ELISA methods demonstrated perfect sensitivity (100%) in detecting celiac disease patients, slightly outperforming the blot test (99% for tTG IgA). This high concordance between different testing methods in our Tunisian cohort is particularly noteworthy, given the genetic heterogeneity of this population as described earlier (2, 3). The high sensitivity of these non-invasive tests in our study supports the findings of Rubio-Tapia et al. (15), suggesting that such tests could potentially reduce the reliance on invasive small intestine biopsies for celiac disease diagnosis in Tunisia (15).

### Implications for clinical practice

The exceptionally high sensitivity of serological tests in our Tunisian cohort suggests that these non-invasive methods could be highly effective in screening for celiac disease in this population. This is particularly relevant given the traditional reliance on small intestine biopsies for diagnosis in Tunisia, as noted by Hariz and colleagues (7). Our findings align with the growing body of evidence supporting non-invasive diagnostic approaches, as discussed in the ESPGHAN guidelines for pediatric populations (25). While our study focused on adult patients, the high accuracy of these tests suggests that they could potentially be used to reduce the need for invasive procedures in carefully selected cases, even in children.

### ASCA, DPG/GAF-3X, PCA, and IF autoantibodies: incidental findings or poly-autoimmunity?

Our data show that ASCA (IgA/IgG) were detectable in a subset of CD patients, whereas positivity in controls was lower. The clinical meaning of ASCA in CD is nuanced. ASCA can arise with increased intestinal permeability and antigen exposure and may be incidental in otherwise uncomplicated CD. In the absence of suggestive symptoms (e.g., chronic diarrhea unresponsive to gluten-free diet, weight loss, abdominal pain, perianal disease), isolated ASCA positivity should not be taken as evidence of inflammatory bowel disease (IBD). Conversely, persistent ASCA – especially at high titers or accompanied by gastrointestinal features atypical for CD – may warrant targeted evaluation for coexisting IBD (26–29).

DGP (deamidated gliadin peptide; including GAF-3X) antibodies are integral to CD pathophysiology and expected to track disease activity; in this context they are not incidental. Their high concordance with tTG and EMA demonstrated in many studies and their reported association with persistent villous atrophy during follow-up support their role as complementary markers rather than indicating a separate autoimmune process (29–31).

By contrast, PCA and IF autoantibodies point to autoimmune gastritis and a risk of pernicious anemia in a subset of patients. In our cohort, PCA were frequent, while IF autoantibodies were rare. These findings could reflect background autoimmunity that sometimes cluster with CD. From a practical standpoint, detection of PCA and/or IF in CD patients should prompt clinical awareness for iron and vitamin B12 deficiency and, where indicated by symptoms and abnormalities (e.g., microcytosis, macrocytosis, low ferritin or B12), further evaluation for autoimmune gastritis (32, 33). Routine endoscopic mapping solely on the basis of low-titer PCA in asymptomatic individuals is not supported by our data; instead, we recommend individualized follow-up anchored to symptoms and hematinic indices.

Together, these patterns align with the concept of poly-autoimmunity, wherein organ-specific autoimmune responses co-occur. Our study was not designed to adjudicate clinical diagnoses of IBD or autoimmune gastritis; thus, we view ASCA, PCA, and IF positivity in this dataset primarily as risk signals that merit context-specific follow-up rather than definitive evidence of comorbid disease.

### Limitations and future directions

Despite the promising results, our study has some limitations.

1. Design and spectrum effects. The case-control design using previously diagnosed celiac disease (CD cases likely inflate apparent accuracy relative to undifferentiated, real-world population (34, 35).
2. Cohort size. The sample is modest (80 CD, 78 disease controls, 80 healthy controls), limiting precision and subgroup analyses.
3. Biopsy confirmation. While duodenal biopsy confirmed CD in serology-positive participants, disease and healthy controls did not undergo biopsy; therefore, occult CD among serology-negative controls cannot be fully excluded.
4. Single-center setting and assay brand. Single-center setting and the use of specific commercial kits may limit generalizability; external validation with alternative platforms is warranted.

## Conclusion

Our study demonstrates exceptionally high sensitivity and specificity of serological tests for celiac disease diagnostics in a Tunisian cohort. These findings suggest that non-invasive serological tests could play a crucial role in celiac disease diagnosis in this population, potentially reducing the need for invasive biopsies. However, further research is needed to fully validate these findings and explore their implications for clinical practice in Tunisia and similar populations.

## Data Availability

The raw data supporting the conclusions of this article will be made available by the authors, without undue reservation.

## Supplementary material

Supplementary Figure 1
**|** Receiver operating characteristics (ROC) curves for ELISA-based detection of celiac disease. **(a)** tTG IgA ELISA; **(b)** DGP ELISA (IgA and/or IgG).
