# Supplementary figures and images for: Prevalence of celiac disease in a Tunisian cohort
Source: Front Gastroenterol (Lausanne). 2025 Oct 20;4:1619533. doi: 10.3389/fgstr.2025.1619533 (PMC12952425; doi:10.3389/fgstr.2025.1619533)

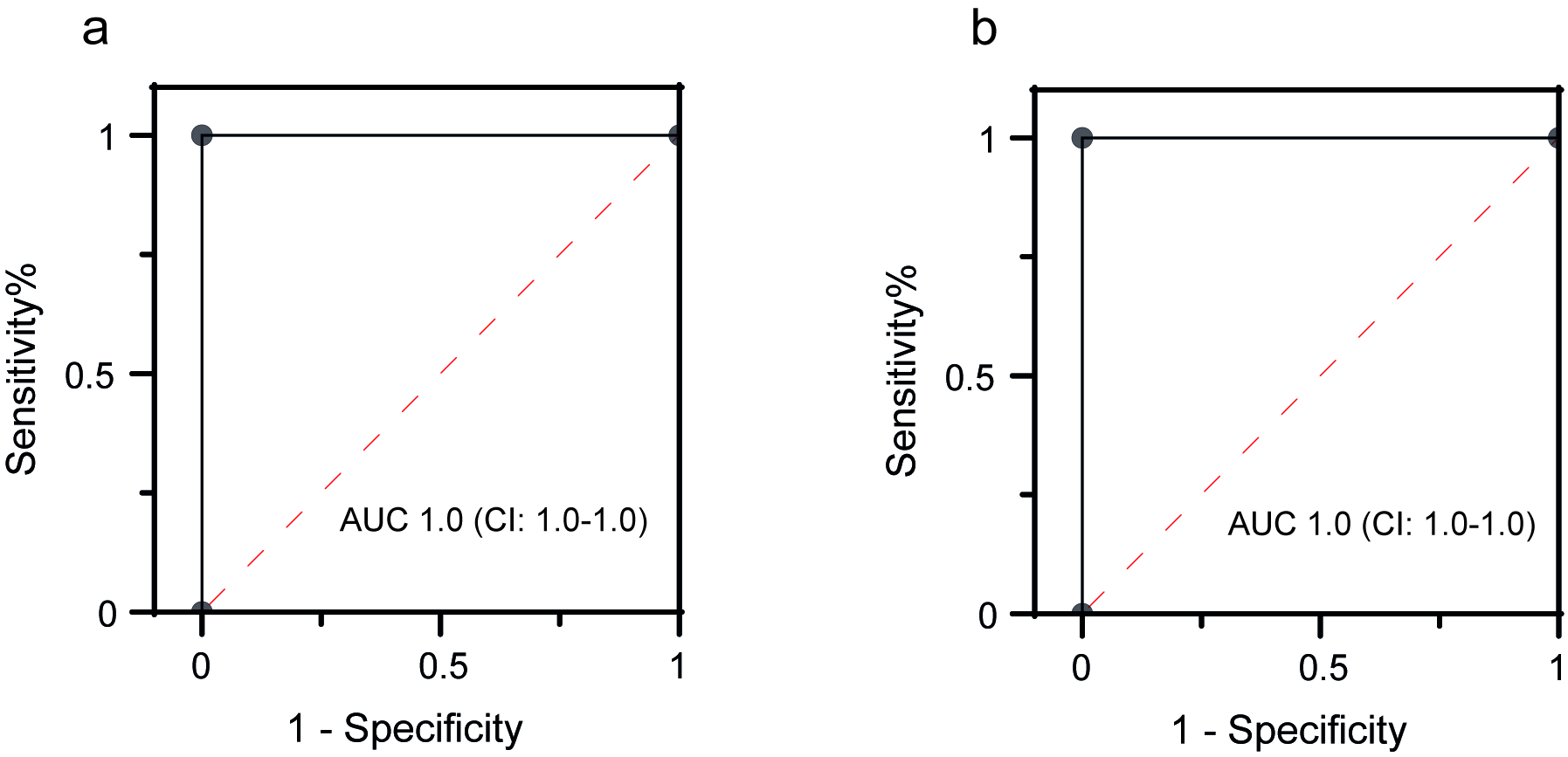

Supplement: Supplementary Figure 1 — | Receiver operating characteristics (ROC) curves for ELISA-based detection of celiac disease. (a) tTG IgA ELISA; (b) DGP ELISA (IgA and/or IgG). [file Image1.tif]
